# Cardiac magnetic resonance feature tracking of the right ventricle in convalescent Kawasaki disease in a large single center

**DOI:** 10.1002/clc.23512

**Published:** 2020-11-12

**Authors:** Qiong Yao, Xi‐hong Hu, Li‐li He

**Affiliations:** ^1^ Department of Radiology Children's Hospital of Fudan University Shanghai China; ^2^ Department of Ultrasound Children's Hospital of Fudan University Shanghai China

**Keywords:** cardiac magnetic resonance, coronary artery aneurysm, feature tracking, Kawasaki disease, myocardial fibrosis, thrombosis

## Abstract

**Background:**

The changes in right ventricular (RV) contractility of Kawasaki disease (KD) still remain unclear.

**Hypothesis:**

We aimed to determine whether RV systolic dysfunction can be detected by cardiac magnetic resonance (CMR) feature tracking and to find its association with coronary artery lesions (aneurysm, thrombosis and stenosis).

**Methods:**

Peak systolic myocardial longitudinal, radial and circumferential strain and the strain rate (RVSL, RVSR, RVSC, RVSRL, RVSRR and RVSRC) in the global RV and three levels (basal, middle and apical) were measured in 66 patients with convalescent KD. A total of 20 controls were included. Comparisons were made with controls and among KD subgroups divided with coronary artery lesions.

**Results:**

RVSC (−10.575% vs. −10.760%), RVSL (−18.150% vs. −18.712%) and RVSRC (−0.815/s vs. −0.924/s) were slightly lower in KD group without significant difference. All the strain and strain rate presented lowest in the basal level. In subgroup comparison, lower RVSL and RVSRL were observed in the giant coronary artery aneurysm (CAA) group; RVSR (15.844% vs. 16.897%), RVSRR (1.245/s vs. 1.322/s) and RVSRC (−0.715/s vs. −0.895/s) were lower in thrombosed group; RVSRL (−1.27/s vs. −1.503/s) were lower in stenosis group. All the comparison in subgroups did not reach significant difference. From the analysis of receiver operating characteristic curve, RVSRL had a better ability to identify KD with giant CAA and stenosis. For the identification of thrombosis, RVSRC had a better ability.

**Conclusions:**

Lower strain and strain rates of RV were detected in convalescent KD. More pronounced in those with persisting coronary artery lesions.

## 1 INTRODUCTION

As a systemic vasculitis of medium sized vessels, Kawasaki disease (KD) is the most common cause of acquired heart disease among children in the developed world. It was reported that coronary artery lesions (CALs) is the dominant common complication, mainly including coronary artery aneurysm (CAA), thrombosis and stenosis. The incidence of CAA is about 5% and huge CAA can usually form thrombosis and ischemic cardiac disease. In the chronic stage, the reconstruction of coronary artery continues and induces stenosis and micro‐flow impairment.

Myocarditis, the most common non‐CAL, accounts for 50%–70% of patients in the acute phase, followed by myocardial fibrosis. For KD, the routine cardiac function often displays normal with routine measurements,^1^ except for patients with ischemic cardiomyopathy and severe CALs.^2^, ^3^, ^4^ However, lower strain has both been detected in the acute and convalescent phase of KD.^5^, ^6^, ^7^ The strain and strain rate are the main indices in the paper. This methodology has the potential to elucidate subtle impairment in myocardial mechanics that cannot be demonstrated by routine cardiac imaging modality.

Compared with echocardiography, cardiac MR feature tracking (CMR‐FT) allows for the quantitative assessment of regional and global myocardial mechanic deformation that is related to myocarditis and myocardial fibrosis. Myocarditis has been found in the entire heart, and the fibrosis continues to the convalescent stage. Previous studies have summarized the left ventricular (LV) myocardial functional impairments in the acute and chronic phase in detail. However, the abnormal mechanics in right ventricle (RV) of KD patients still remains undefined. This is the first study to apply CMR‐FT in KD to examine mechanic deformation in RV and to discover its association with CALs. Our goal was to define the variation of RV myocardial deformation within KD cohorts and to assess if variation in myocardial deformation may help identify subgroups with higher risk.

## 2 METHODS

### 2.1 Patients and controls

The study was conducted in Children's Hospital of Fudan University in Shanghai, China. For patients with KD, when they were admitted into the hospital, the first exam was echocardiography. After finding CALs on echocardiography, CMR was performed for myocardial and coronary artery evaluation and digital subtraction angiography (DSA) was conducted as a golden standard for CALs. From May 2013 and May 2020, totally 78 KD patients with CMR were collected in our center. We reviewed all the images and chose 66 patients in our cohort to evaluate the RV contractility impairment. We excluded five patients without good CMR imaging quality to evaluate the CALs, three patients without adequate CMR sequence for strain analysis, and two cases without DSA to confirm the CALs. A total of 20 cases with matched age and sex were enrolled in the control group. The study was approved by the Ethics Committee of our hospital and all patients signed written informed consents. The phases were defined as follow: acute phase, day 1–14, subacute phase, day 15–42, and convalescent/chronic phase, after day 43.^7^

### 2.2 Cardiac magnetic resonance protocol

All patients and controls experienced CMR at 1.5‐T MR (Siemens Medical Solutions, Erlangen, Germany). Imaging was conducted under oral sedation for the patients under 8‐year‐old or who could not cooperate to complete the CMR.

A combination of 16‐elements phased array surface coil and spine coil were used. Initially, a survey examination in three orthogoal planes was performed to localization of the heart, and then an interactive sequence was performed to define patient‐specific relevant orientations (including axial, coronal, sagittal, two‐chamber, four‐chamber and short axial views). Electrocardiography (ECG)‐gated two‐dimensional steady‐state free precession (SSFP) segmented cine images in short axial and longitude plans with full coverage of the LV and RV were acquired for functional assessment and CMR‐FT assessment. The imaging parameters were listed below: field‐of‐view (FOV) = 240 × 240 mm^2^, repetition time (TR) 48–84 ms, temporal resolution 50 ms, flip angle 62°, voxel size 1.2 × 1.2 × 6 mm^3^. The images were acquired during free breathing with navigator triggering for respiratory compensation.

A 3D volume of axial images covering the heart and aortic arch were acquired using SSFP sequence (TR 294 ~ 337 ms, flip angle 90°, 1 mm slice thickness, FOV = 164 ~ 380 × 250–420 mm^2^) with T2 preparation to suppress the signal from the myocardium and fat suppression to suppress the high signal from epicardial fat. The whole heart and the CALs in the main coronary arteries were viewed in this sequence, including the right coronary artery (RCA), the left coronary artery (LCA), the left anterior descending artery (LAD), the left circumflex artery (CCX).

### 2.3 CMR analysis

**FIGURE 1:**
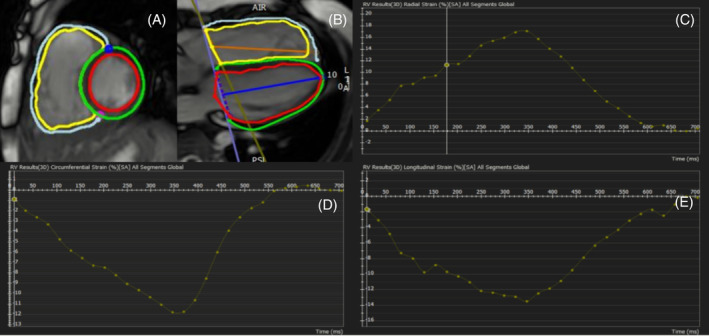
CMR‐FT post processing by CVI42 software. Delineation of endocardial and epicardial borders of the left and right ventricles in end‐systolic and end‐diastolic phases in the axial short (A) and longitude (B) cine images for strain and strain rate calculation. Global right ventricular radial strain (C), circumferential strain (D), and longitudinal strain (E) curve in one cardiac cycle were produced. CMR‐FT: Cardiac magnetic resonance feature tracking

The analysis of CMR‐FT was performed by axial short and longitude cine images with commercial software Circle (CMR42, version 5.6.5, Cardiovascular Imaging Inc., Calgary, Alberta, Canada), as previously described.^7^ Global and regional strain analyses of the RV free wall in three directions (longitudinal, circumferential and radial) were measured in basal, middle and apical levels. The systolic strain and strain rate of LV and RV were calculated by tracing endocardial and epicardial borders of both ventricles in end‐systolic and end‐diastolic phases. Contours were manually corrected when automatic tracking was not accurate enough. Examples of contours for CMR‐FT and the typical strain output curves were shown in Figure 1.

The quantification of the LV and RV volume indices were normalized to the body surface area. The 3D SSFP images were applied to evaluate the presence of aneurysms, stenosis and thrombi of RCA. According to 2017 AHA standard^8^ based on z score, CAA was defined as small CAA (≥2.5–<5), medium CAA (≥5–<10) and giant CAA (≥10).

All the CMR studies were analyzed by 1 radiologist who was blinded to the patient clinical data. In addition, random samples of 20 cases were re‐evaluated by the same radiologist to assess intra‐observer and by another investigator for inter‐observer agreement. There exist no consensus normal values for myocardial deformation in children, so we compared the KD group with the control group.^9^

### 2.4 Statistical analysis

All data were reported as mean ± SD. Unpaired Student's *t*‐test and one‐way ANOVA were adopted to compare the strain and strain rate between subgroups. Diagnostic accuracy of CMR parameters was evaluated through receiver operating characteristic (ROC) curve analysis. For area, under the curve (AUC), a value of 0.9–1.0 was considered excellent, 0.75–0.9 marked good, 0.6–0.75 stood for moderate and 0.5–0.6 was poor. Best cut‐off values, sensitivity and specificity were derived from ROC curves. Intra‐ and inter‐observer variability was assessed using Cronbach's α. Statistical analysis was performed with SPSS software (SPSS Inc., Chicago, IL, version 26.0). A *p*‐value of ≤.05 was regarded statistically significant.

## 3 RESULTS

### 3.1 Demographic information of patients and controls

In Table 1 the demographic of the KD patients and controls were summarized. All KD patients met the diagnostic criteria for KD and received optimal treatment including intravenous immunoglobulin after diagnosis. A reference control group of 20 healthy volunteers with no evidence of cardiac disease were identified for comparison.

**TABLE 1:** Demographic information of KD patients

Sex	
Male	53
Female	13
Age of onset (month, IQR)	29.673 ± 22.256 (7–43)
Interval of exam (month, IQR)	19.980 ± 16.664 (2–36)
CMR parameters	
LVEF (%)	68.950 ± 5.726
LVEDVi (ml/m^2^)	83.348 ± 20.244
LVESVi (ml/m^2^)	22.891 ± 12.632
RVEF (%)	62.787 ± 7.410
RVEDVi (ml/m^2^)	81.179 ± 10.565
RVESVi (ml/m^2^)	19.588 ± 5.429
CAA in RCA	
Small	12/66 (18.182%)
Median	29/66 (43.939%)
Giant	15/66 (22.727%)
Thrombosed	13/66 (19.697%)
Stenosis	4/66 (6.060%)

Abbreviations: CAA, coronary artery aneurysms; IQR, inter‐quartile range; KD, Kawasaki disease; LVEDVi, left ventricular end‐diastolic volume index; LVESVi, left ventricular end‐diastolic volume index; LVEF, left ventricular ejection fraction; RCA, right coronary artery; RVEF, right ventricular ejection fraction; RVEDVi, right ventricular end‐diastolic volume index; RVESVi, right ventricular end‐diastolic volume index.

### 3.2 Cardiac magnetic resonance results

**FIGURE 2:**
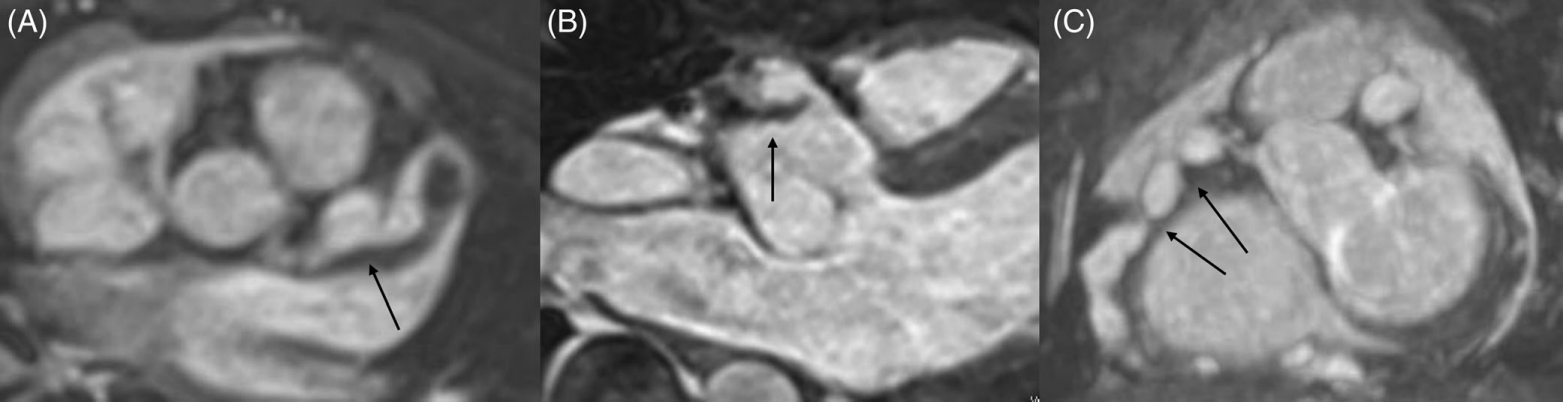
A, A 2‐year‐old girl with a giant CAA (arrow) in the proximal segment of LAD. B, A giant aneurysm in the proximal segment of RCA associated with mural thrombus (arrow), presenting higher signal on 3D‐SSPF images. C, Multiple CAAs were found along the whole RCA and the stenosis could be found between the proximal giant CAAs (arrow). CAA: coronary artery aneurysm; LAD: left anterior descending artery; RCA: right coronary artery; SSPF: steady‐state free precession

Totally, 66 CMR exams were carried out and routine CMR indices of RV cardiac function were normal, without any wall motion abnormality (Table 1). A total of 56 patients had CAAs in RCA, of which15 suffered from giant CAAs that were mainly in the proximal segment of RCA, followed by the middle segment. CMR detected thrombus formation in RCA in 13 patients, and RCA stenosis in four patients. Thirty CAAs were formed at the LCA branching site extending to the LAD; eight CAAs developed at the branching site of the LAD and CCX. Figure 2 showed the CALs detected by 3D SSFP images. All the CALs detected on CMR were highly consistent with DSA. For subgroup analyses, patients were subdivided according to CALs in RCA, such as the z‐score of CAA, the existence of thrombosis and stenosis.

### 3.3 CMR‐FT of RV in KD group versus controls

Compared with controls, RVSC, RVSL and RVSRC were slightly lower in KD group (Table 2). The above differences did not reach statistical significance. Among the three levels (apical, middle and basal), all the lowest strain and strain rates were observed in the basal segment, which reached statistical significance (Table 3).

**TABLE 2:** Comparison of RV strain and strain rate between healthy volunteers, KD patients and subgroups

	KD	Controls	No CAA	Small CAA	Median CAA	Huge CAA	Stenosis (+)	Stenosis (−)	Thrombsis (+)	Thrombosis (−)
	(n = 66)	(n = 20)	(n = 10)	(n = 12)	(n = 29)	(n = 15)	(n = 4)	(n = 62)	(n = 13)	(n = 53)
RVSR (%)	15.820 ± 9.680	14.501 ± 3.093	14.603 ± 8.075	16.613 ± 8.192	16.108 ± 7.783	19.266 ± 12.055	17.810 ± 4.409	16.617 ± 9.184	15.844 ± 5.822*	16.897 ± 9.598
RVSC (%)	−10.575 ± 7.6375*	−10.760 ± 2.758	−9.44 ± 4.663	−10.691 ± 4.394	−10.314 ± 4.196	−11.885 ± 5.132	−11.078 ± 3.043	−10.577 ± 4.584	−10.384 ± 3.473	−10.662 ± 4.733
RVSL (%)	−18.150 ± 13.055*	−18.712 ± 5.798	−21.722 ± 9.37	−18.132 ± 9.904	−19.148 ± 8.172	−16.587 ± 10.303*	−20.273 ± 6.552	−18.674 ± 9.281	−21.575 ± 7.294	−18.083 ± 9.433
RVSRR (1/s)	1.150 ± 1.683	1.098 ± 0.821	1.138 ± 0.589	1.475 ± 0.808	1.281 ± 0.551	1.334 ± 0.644	1.298 ± 0.388	1.307 ± 0.638	1.245 ± 0.479*	1.322 ± 0.657
RVSRC (1/s)	−0.815 ± 0.615*	−0.924 ± 0.897	−0.761 ± 0.339	−0.982 ± 0.444	−0.833 ± 0.494	−0.880 ± 0.320	−0.900 ± 0.277	−0.857 ± 0.435	−0.715 ± 0.606*	−0.895 ± 0.368
RVSRL (1/s)	−1.540 ± 1.085	−1.409 ± 0.499	−1.072 ± 2.903	−1.929 ± 0.633	−1.552 ± 0.897	−1.294 ± 0.557*	−1.27 ± 0.442*	−1.503 ± 1.35	−1.657 ± 0.594	−1.448 ± 1.436

*Note:* *presenting lower value without statistical significance.

Abbreviations: CAA, coronary artery aneurysms; KD, Kawasaki disease; RVSC, right ventricular strain circumferential; RVSL, right ventricular strain longitudinal; RVSR, right ventricular strain radial; RVSRC, right ventricular strain rate circumferential; RVSRL, right ventricular strain rate longitudinal; RVSRR, right ventricular strain rate radial.

**TABLE 3:** Comparison of RV strain and strain rate among three levels

Location	RVSR (%)	RVSC (%)	RVSL (%)	RVSRR (1/s)	RVSRC (1/s)	RVSRL (1/s)
Basal	15.610 ± 14.797*	−6.329 ± 13.152*	−15.664 ± 6.695*	1.196 ± 1.646*	0.558 ± 1.374*	−1.240 ± 1.085*
Middle	17.632 ± 8.699	−10.306 ± 6.432	−18.150 ± 13.055	1.381 ± 1.007	0.942 ± 0.714	−1.540 ± 1.085
Apical	21.392 ± 13.576	−12.858 ± 6.196	−20.150 ± 13.055	1.782 ± 1.162	1.031 ± 0.967	−1.740 ± 1.085
*p* value	.030	.000	.000	.032	.025	.000

Abbreviations: RV, right ventricular; RVSC, right ventricular strain circumferential; RVSL, right ventricular strain longitudinal; RVSR, right ventricular strain radial; RVSRC, right ventricular strain rate circumferential; RVSRL, right ventricular strain rate longitudinal; RVSRR, right ventricular strain rate radial. *p < 0.05.

### 3.4 CMR‐FT comparisons in KD subgroups

Comparisons were made between KD subgroups subdivided by z score of CAAs in the RCA. Among 66 patients, 10 did not experience CAA in the RCA, 12 with small CAA, 29 with median size of CAAs, and 15 with giant CAAs. When KD patients were subdivided into four groups according to z score of CAA, lower RVSL, RVSRC and RVSRL were observed in the giant CAA group. However, no statistically significant difference was illustrated among the subgroups (Table 2).

When we compared patients suffering from thrombi (n = 13) with those without thrombi (n = 53), RVSR, RVSRR and RVSRC were lower in thrombosed group, but did not reach statistical difference.

When patients catching with stenosis (n = 4) were compared with those without stenosis (n = 62), RVSRL were lower in stenosis group, but did not reach statistical significant difference.

### 3.5 ROC analysis

ROC curve revealed that RVSRL had a better ability to identify KD from giant CAA and stenosis. The cut‐off value for RVSRL was −1.405 (sensitivity = 0.489, specificity = 0.737) and −1.15 (sensitivity = 0.750, specificity = 0.758) respectively. For the identification of thrombosis, RVSRC had a better ability with a cut‐off of −0.565 (sensitivity = 0.385, specificity = 0.830). The results were listed in Table S1.

### 3.6 Inter‐ and intra‐observer agreements

Twenty subjects were randomly selected from KD group to assess inter‐ and intra‐observer agreement of CMR‐FT analysis. We evaluated the intra‐observer and inter‐observer agreement by Cronbach's α. If the value is higher than 0.8, the reliability is high. If the value is between 0.7 and 0.8, the reliability is good. If the value is between 0.6 and 0.7, the reliability is acceptable. If the value is less than 0.6, the reliability is poor. According to CMR‐based strain parameters, we identified the good reliability in the inter‐ and intra‐observer agreement, except for the inter‐observer agreement of RVSRR (Cronbach's α = 0.651).

## 4 DISCUSSION

As a systemic vasculitis of medium sized vessels, perhaps the pericardium, myocardium, valves and the coronary arteries were inflamed in KD.^10^ Myocardial inflammation is the most common noncoronary cardiac abnormalities and has been found in about 50%–70% patients in acute phase. Biopsy studies suggested that the myocarditis ranged from the regional segments to the entire heart.^11^, ^12^ Myocarditis in KD may improve rapidly and the long‐term sequelae includes hypertrophy and interstitial fibrosis. For children with KD, decreased ventricular contractility may be resulted by myocardial inflammation and fibrosis in the acute and chronic stage, even with normal cardiac function. For RV, the myocardium is too thin and CMR cannot analyze the myocardial impairment accurately, including the edema, fibrosis and ischemia. We cannot prove the existence of myocarditis in RV directly. Meanwhile the areas of myocarditis were not only confined in the areas with giant CAAs. But for the patients with giant CAAs, they usually have higher inflammatory biomarkers and more severe micro‐ and macro‐vascular impairments, inducing more severe myocarditis. In the chronic stage of KD, more severe myocardial ischemia and interstitial fibrosis have been identified. So we think there are correlations between CALs and RV strain impairment and made the analysis.

It has been proved that the strain analysis was superior to routine cardiac function for evaluation of subclinical LV systolic dysfunction in many diseases, including KD.^13^, ^14^ It was obvious that in KD, LV myocardial strain and strain rate were reduced despite the normal LV systolic function.^1^, ^15^, ^16^ RV myocardial strain, and both global and segmental strains, have also been found impaired in many clinical scenarios, such as congenital heart disease, pulmonary hypertension and heart failure.^17^, ^18^ In a research of 364 patients, the strain analysis by CMR‐FT was significantly associated with future cardiac events, especially the RV global radial strain and LV global transverse strain^19^. In researches of repaired TOF patients, reduced strain values were related with cardiac function and functional capacity of the cardiopulmonary exercise test.^20^ RCA is often affected in KD, but the researches of RV contractility are limited. Based on a recent data, the global and regional RV strain values of KD patients were decreased compared with control subjects.^7^ However, in another research, including 15 KD patients, there was no significant difference in the RV global longitudinal strain and strain rate.^16^ Overall, the existing evidence about myocardial deformation studies of RV in KD is poor.

In our study, a considerable proportion of patients suffer from normal EF and abnormal RV strain indices, indicating that myocardial mechanic deformation impairment starts before RV cardiac dysfunction. KD patients were revealed to have lower RVSC, RVSL and RVSRC compared with age‐matched normal controls. The results did not reach statistic difference, which may be caused by the preserved EF in this group. Meanwhile, we also found that the proximal segments presented more obvious strain abnormality, which was related with the CAAs that mostly existed in the proximal segments of RCA. The paper had unraveled the non‐uniform mechanics of RV, characterized by increased strain values at the apex.^21^ That would also contribute to the increase of strain and strain value of the middle and apex level.

In subgroup analysis, a more pronounced decrease was shown in those with persisting CALs versus those without persisting CALs.^7^ The presence of CALs is considered the most common complication of KD, such as dilation, CAA, stenosis, occlusion and thrombosis formation. As a supportive diagnosis of KD, the prevalence of CAA reduced from 23% to 8% after using IVIG. CAA variates in numbers and sizes, usually locating in proximal segments and then extending to distal part (very rare). Cardiovascular complications, thrombotic occlusion and severe stenosis occur mainly in giant CAA and can cause myocardial infarction.^22^, ^23^ Myocarditis and interstitial fibrosis are more frequent for patients experiencing large CAA^24^, ^25^ and had higher inflammatory biomarkers.^26^, ^27^, ^28^ Researchers have found the LV myocardial strain impairment with CALs.^1^, ^9^, ^29^ In this research, it was discovered that the RVSRL was more sensitive for giant CAA and stenosis detection, compared with other indices. It is plausible that the RV fibers are predominantly arranged along the longitudinal axis, and the longitudinal myocardia layer mostly leads to RV shortening.^30^ So the impairment of longitude strain was the most obvious one in KD. Meanwhile, the systolic strain rate was regarded as the a more robust parameter, reflecting myocardial function, rather than strain,^31^, ^32^, ^33^ because strain may be affected by interstitial inflammation/edema, while strain rate may not be affected unless that actual myocyte injury occurs. The results of this study offer additional insights into the value of the early detection of RV contractility deterioration as an independent predictor of more severe CALs and myocardial fibrosis.

For echocardiography, currently, the qualitative evaluation of RV cardiac function is a diagnostic challenge due to the unusual shape and uneven contractility pattern.^34^ CMR has been the gold standard method in RV function evaluation, and FT‐CMR can add value for RV contractility abnormalities.^34^, ^35^, ^36^, ^37^, ^38^ Reproducible and repeatable quantification of RV strain indices are also vital to monitor patients.^39^ The analysis of RV function by FT‐CMR was feasible and reproducible.^31^, ^40^ In our research, the reproducibility was good, as reported in other papers.^41^ Moreover, the CVI 42 software also proved excellent intra‐ and inter‐observer reproducibility for RV regional strain values.^31^, ^41^, ^42^ The good reproducibility, inter‐reader and intra‐reader variability, make FT‐CMR a robust tool to support decision‐making and follow‐up.^43^ Meanwhile, CMR can afford the assessment of myocardial perfusion, scarring in a single examination, as well as the evaluation of regional wall motion.^33^, ^44^

Although the number of patients in this study was large enough, it still existed several limitations. (1) We did not include KD children without CALs, so we missed the data of this subgroup. (2) Because of the difficulty of assessment to the thin‐walled RV, the evaluation of RV myocardial perfusion and scarring was not conducted. (3) The accuracy of CMR and echocardiography of strain analysis was not compared. Therefore, future studies should be conducted to solve these problems.

## 5 CONCLUSION

In KD, a reduction in strain of RV was observed in the convalescent stage, detecting subclinical functional abnormalities. A more pronounced decrease was displayed in those with persisting CALs. These results provide new insight into the effects of strain analysis on ventricular mechanics and may prompt modifications in management.

## CONFLICT OF INTEREST

The authors declare that they have no competing interests.

## ETHICS APPROVAL

Informed consent forms were signed by the parents and ethics approval was approved by the institutional review boards of the Children's Hospital of Fudan University.

## Supporting information


**Table S1.** AUC of RV strain and strain rate parameters for detection of huge CAA, thrombosis and stenosis. AUC = aera under curve; CAA = coronary artery aneurysms; RVSR = right ventricular strain radial; RVSC = right ventricular strain circumferential; RVSL = right ventricular strain longitudinal; RVSRR = right ventricular strain rate radial; RVSRC = right ventricular strain rate circumferential; RVSRL = right ventricular strain rate longitudinal. *presenting the item with highest AUC.

## Data Availability

The data that support the findings of this study are available from the corresponding author upon reasonable request
