# Supplementary material for: Cardiac magnetic resonance feature tracking of the right ventricle in convalescent Kawasaki disease in a large single center
Source: Clin Cardiol. 2020 Nov 12;44(1):108–15. doi: 10.1002/clc.23512 (PMC7803351; doi:10.1002/clc.23512)
Supplement: Supplementary file 1 — Table S1. AUC of RV strain and strain rate parameters for detection of huge CAA, thrombosis and stenosis. AUC = aera under curve; CAA = coronary artery aneurysms; RVSR = right ventricular strain radial; RVSC = right ventricular strain circumferential; RVSL = right ventricular strain longitudinal; RVSRR = right ventricular strain rate radial; RVSRC = right ventricular strain rate circumferential; RVSRL = right ventricular strain rate longitudinal. *presenting the item with highest AUC. [file CLC-44-108-s001.docx]

Supplementary Table 1: AUC of RV strain and strain rate parameters for detection of huge CAA, thrombosis and stenosis. AUC = aera under curve; CAA = coronary artery aneurysms; RVSR = right ventricular strain radial; RVSC = right ventricular strain circumferential; RVSL = right ventricular strain longitudinal; RVSRR = right ventricular strain rate radial; RVSRC = right ventricular strain rate circumferential; RVSRL = right ventricular strain rate longitudinal. ^✳^presenting the item with highest AUC

|  | Huge CAA | Thrombosis | Stenosis |
| --- | --- | --- | --- |
| RVSR (%) | 0.615 | 0.538^✳^ | 0.619 |
| RVSC (%) | 0.399 | 0.470 | 0.444 |
| RVSL (%) | 0.458 | 0.397 | 0.456 |
| RVSRR (1/s) | 0.577 | 0.500 | 0.552 |
| RVSRC (1/s) | 0.424 | 0.551 | 0.456 |
| RVSRL (1/s) | 0.679^✳^ | 0.462 | 0.667^✳^ |
